# Investigating the prevention and mitigatory role of risk communication in the COVID-19 pandemic: A case study of Bloemfontein, South Africa

**DOI:** 10.4102/jamba.v13i1.1130

**Published:** 2021-09-27

**Authors:** Olivia Kunguma, Mosekama O. Mokhele, Mercia Coetzee

**Affiliations:** 1 Disaster Management Training and Education Centre for Africa, Faculty of Natural and Agricultural Sciences, University of the Free State, Bloemfontein, South Africa; 2 Department of Organisation Sciences, Vrije University, Amsterdam, the Netherlands; 3 Department of Communication Science, Faculty of Humanities, Central University of Technology, Bloemfontein, South Africa

**Keywords:** COVID-19, risk communication, disaster management, legislation, policy, media, disaster communication, Agenda-setting Theory

## Abstract

The South African disaster response activities surpass risk reduction since the implementation of the *Disaster Management Act* 57 of 2002 (DMA) and the *National Disaster Management Framework* of 2005 (NDMF). Risk reduction, in particular risk communication, remained unexploited until the occurrence of coronavirus disease 2019 (COVID-19) pandemic. The legislation and policy mandate a proactive approach for disaster management, requiring a focus on disaster risk reduction. Therefore, this study aimed to assess the significance of risk communication as a critical prevention and mitigatory strategy in disaster risk management, focusing on the COVID-19 pandemic. Key to risk communication success is ensuring adequate comprehension, accurate perception of the disseminated information, and compliance with regulations. Questions of trustworthiness, acceptability, effectiveness, and usefulness of messages and strategies communicated sought answers from the Bloemfontein population. Furthermore, the Agenda-setting Theory provided the grounding for the study. The study sample was picked in a stratified random sampling manner, using the confidence level and margin of error equation. A questionnaire survey was used to collect the data required to achieve the research objectives. Risk communication as a disaster risk reduction strategy implemented concurrently with imposed regulations was found to have played a vital role in mitigating the virus spread. However, the respondents were not aware of the local disaster management centre, which is supposed to be engaged in COVID-19 disaster management activities.

## Introduction

Amidst the pandemonium of developing a coronavirus vaccine, preventing and mitigating the spread of coronavirus disease 2019 (COVID-19), risk communication proved by far to be one of the profound methods of containment (South African Government News Agency 2020; World Health Organization [WHO] 2020a). World leaders realised that they are not at war with the virus but with the human factor, which involves analysing how human beings behave physically and psychologically in response or relation to a particular situation (Rouse 2020). In this case, world leaders and health officials made a call to limit the spread of COVID-19 through relaying robust preventative and mitigative measures. At the same time, they faced the challenge of understanding how people perceive the virus and how to change certain behaviours to combat the virus.

Since WHO declared COVID-19 a global pandemic, most countries were tasked with implementing strategies to deal with the alarming levels of the spread of the virus and its severity (WHO 2020b). In South Africa, the National Disaster Management Centre (NDMC) was proactive in its approach, immediately classifying COVID-19 as a national disaster (Republic of South Africa 2020a). Following the NDMCs classification, the Minister of Cooperative Governance and Traditional Affairs (CoGTA)^1^, declared COVID-19 a national disaster. Accordingly, the President of South Africa acceded to these directives and on 15 March 2020, announced the declaration of the COVID-19 pandemic as a national disaster (Republic of South Africa 2020b; Reyburn 2020).

Since the COVID-19 national disaster declaration was issued, risk communication, an essential component of disaster risk reduction, revealed its significance in the disaster management sector. Important to note is that Van Niekerk (2014) and Botha et al. (2011), found that not all disaster managers in South Africa integrate disaster risk reduction in development planning; they attributed this deficiency to their lack of disaster risk reduction comprehension. Nevertheless, in an event where disaster managers implemented disaster risk reduction strategies, the risk communication component was minimally exploited, if not at all. Scholars like Chagutah (2014), Van Niekerk (2014) and Humby (2012), among others, found that disaster managers’ communication and public awareness strategies concerning risk and risk reduction are meagre. Furthermore, it was found that communities’ response to the few risk reduction strategies implemented is mostly insignificant. Chagutah (2014) and Wisner et al. (2004), postulate that in most cases, communities do not trust the risk communicators and perceive proposed strategies as threats to their livelihoods. As a result, these community members resist changing their behaviours towards the communicated risk avoidance strategies.

With that in mind, this research sought to investigate the role risk communication played in mitigating and preventing the spread of COVID-19 in Bloemfontein, South Africa, from a disaster management perspective. As the role of risk communication is essential, the effectiveness thereof in changing behaviour towards risk avoidance is important, and so is the comprehension of the messages communicated. Significance in all of this is the communication mediums used, the trustworthiness of the risk communicators, the acceptability and feasibility of strategies implemented, and compliance with the regulations. Also, the communities knowledge of institutions at the local level, in particular, the disaster management centre, which plays a significant role in preventing and mitigating the virus was investigated. As part of the literature, the Agenda-setting Theory (AST) variables were explored to understand the aspects under investigation.

### Significance of risk communication

The deadly effects of a disaster can be reduced by implementing the following vital strategies: anticipating, educating, and informing those at risk (Rambau, Beukes & Frazer 2012). The WHO (2012), defines risk communication in line with the postulations of Rambau et al. (2012), stating that risk communication is the exchange of real-time information, advice, and opinions between experts and the people facing threats to their health, economic, and social well-being. This kind of communication aims to enable people at risk to make informed decisions to protect themselves and their families. Risk communication shapes people’s perceptions of risk and influences their disaster preparedness and response actions. Therefore, risk communication is an important component of disaster risk management (Shaw et al. 2013).

Key to the effectiveness of risk communication is ensuring adequate comprehension, accurate perception of the disseminated information, and the use of trusted sources of information (Chimusoro et al. 2018). Besides, risk communication aims to share information to change beliefs and behaviour (Naik, Ashmed & Edwards 2012). Another dimension of risk communication is the feasibility of the actions recommended and the population’s ability to apply these actions (Glik 2007). Governments can activate emergency response mechanisms that aim to reduce the risk at hand, in this case, COVID-19. Risk reduction through risk communication is therefore based on a community’s perceptions, concerns, and beliefs. Also, their knowledge and practices in identifying rumours and misinformation during an emergency.

Globally, the increase in disaster declarations has subsequently augmented the significant need of disseminating appropriate and relevant risk information. Historical epidemics, for example, the 2008 and 2009 cholera outbreak in Zimbabwe, reveal the benefits of risk communication. Chimusoro et al. (2018) found that despite the economic challenges Zimbabwe faced during the epidemic, the country was able to contain the cholera outbreak through dissemination of risk communication messages and containment strategies. In the face of the COVID-19, countries such as Singapore, South Korea, and Rwanda put in place mitigatory strategies to curb the spread of the virus, which includes the execution of effective risk communication. In Singapore, a combination of factors such as, top-class health systems, tracing and containment measures, and effective communication contributed to controlling the virus spread (Heijmans 2020). South Korea contained COVID-19 without a lockdown, one of the drastic regulations imposed by most countries. South Korea was swift and aggressive with mass testing through drive and walk-through testing stations and vigorous contact tracing. However, South Korea mostly attributed its success to social capital, volunteerism, the Korean people’s compliance, and the government’s proactiveness, openness, and credibility (Investec 2020). In Africa, Rwanda was also prompt in its response to the first case of COVID-19: 5 days after the first case was reported, they controlled the spread of the virus through tracing, isolation, testing, and lockdown (Moore 2020).

In comparing South Africa to these countries, the researchers realised that Singapore was probably successful because their measures were more inclusive than those of South Africa. The Singaporeans adapted better to the COVID-19 restrictions as compared to the African countries (Kuguyo, Kengne & Dandara 2020). Besides, South Africa’s health system is flawed compared to Singapore’s health standards. South Africa and South Korea have similarities in the measures employed, but even though South Africa went into lockdown only 10 days after the first report of a positive case on 5 March 2020 (Mdeni 2020), it still experiences difficulties in controlling the spread of the virus. The low compliance in South Africa because of poverty could be the reason for the difficulties in controlling the spread of the coronavirus (Kuguyo et al. 2020).^2^ Despite the overwhelming regulations and risk messages communicated and their success in controlling the virus in some countries, South Africa is yet to see adherence to these strategies before the success thereof can be measured.

Some population groups in South Africa disputed some of the COVID-19 strategies imposed because of the challenges they presented. Amongst several disputed challenges, social distancing was one of them; because of the informal settlement residential status of some South Africans being a prime reason behind it (Nyashanu, Simbanegavi & Gibson 2020). Regardless of the challenges, South Africa communicated many risk communication messages and regulations (see Table 1) since the national disaster declaration on 15 March 2020. Some of the regulations announced were to stay enforced during the national state of disaster and some of them have since been revoked or changed (Republic of South Africa 2020c).

**TABLE 1:** South African risk communication messages and regulations.

Variable	Examples of COVID-19 risk communication messages and regulations
Risk communication messages	Wash your hands regularly with soap or an alcohol-based hand sanitiser.
	Avoid touching your eyes, nose, and mouth with unwashed hands.
	Avoid close contact with sick people.
	Cover your mouth and nose when you cough or sneeze with a flexed elbow or a tissue, then throw the tissue in the bin.
	Practice social distancing of 1.5 meters away from other people.
	Self-quarantine.
	Clean and disinfect frequently touched objects and surfaces.
Regulations	All stakeholders to release the resources required to help combat and manage the virus spread.
	Prevent and prohibit gatherings of more than 50 people.
	A person who tested positive or suspected of contracting COVID-19 may not refuse medical examination, treatment, isolation, or quarantine.
	Closure of schools from 18 March 2020
	Suspend visits to detention centres and shelters.
	Limit the sale and transportation of alcoholic beverages.
	Any refusal to abide by the regulations calls for a fine or imprisonment of 6 months or both.
	Lockdown was issued on 23 March 2020 (emergency protocol requiring everyone in South Africa to stay at home except for essential purposes like grocery stores, pharmacies, social grant collection, and banks).

*Source*: Department of Health (DoH), 2020, *COVID -19 corona virus South African resources portal*, viewed from https://sacoronavirus.co.za/; Republic of South Africa, 2020c, *CoGTA Minister gazettes regulations to deal with COVID-19 virus outbreak*, viewed from http://www.cogta.gov.za/?p=7752.

COVID-19, coronavirus disease 2019.

### Coronavirus disease 2019: Role of the Local Disaster Management Centre

The Mangaung Metropolitan Disaster Management Centre is the local disaster management centre, which is based in Bloemfontein. It coordinates disaster events at the local level and supports the provincial and NDMCs. During the pandemic, their expected role was to coordinate departments like the South African Police Services (SAPS) and the Department of Health. They also had to ensure compliance with the COVID-19 regulations at public gatherings such as funerals or church gatherings. Other roles were the monitoring of infection progression, development of reports, public awareness and serving on various COVID-19 committees. Since the disaster management institution is a primary stakeholder in responding to COVID-19, their visibility at the local level in ensuring adherence to risk communication is important. Ther were also entrusted with improving the trustworthiness and credibility of the risk communication initiatives

### Challenges with the coronavirus disease 2019 risk messages and regulations imposed

Given the nature and the living dynamics of some South African citizens such as culture, beliefs, norms, living environment, and economic status, these may hinder the consumption of the risk messages communicated and regulations imposed. In turn, the circumstances for example the lack of running water instil receptivity resistance or challenges among many community members. Receptivity resistance to COVID-19 is when an individual is not able and willing to take in information or an idea. Based on the National Hand Hygiene Behaviour Change Strategy 2016–2020, a 2012 General Household Survey found that about 11% of South African households have inadequate sanitation services. It was also found that 26% of households have deteriorated sanitation infrastructure (DoH 2017; UNICEF 2017). Additionally, the survey discovered that most South Africans live in settlements exposed to a high risk of contracting water, sanitation, and hygiene-related diseases. Khayelitsha in Cape Town is one of the areas in South Africa that do not have access to sufficient water and sanitation facilities. The residents live in fear of contracting the virus because they have received the ‘wash your hands for 20 seconds’ risk communication message, but the lack of access to water affects the call to action (Lali et al. 2020).

In Bloemfontein, high-density suburbs such as Batho and Bochabela have blocked and broken sewerage pipes. The water quality is poor and in most informal settlements there is limited access to water (Pretorius & DeVilliers 2002). In a 2016 Community Survey, at least 52% of households have piped water inside the yard, 39% receive piped water inside the dwelling, 4% receive piped water on a community stand, 2% in a community tap, and the rest obtain water from elsewhere (Cooperative Governance & Traditional Affairs 2020). These results show that at least the water situation in Bloemfontein is not a deterring factor when it comes to responding to COVID-19. This is when, South Africa, in its entirety, faces water challenges because of failing infrastructure as well as water shortages caused by drought in the last few years. These difficulties left the government concerned about the spread of the COVID-19 virus (Harrisberg & Peyton 2020).

Risk messages communicating social distancing might prove difficult amongst communities that are family-oriented and practice a collectivist culture. Lali et al. (2020) found that social distancing poses challenges because respondents in their study said: ‘The virus makes me scared because we have also been told to limit kisses, while in our culture we often greet and kiss others’. People’s collectivist culture might also affect the regulation of isolation, quarantine, and distancing from sick people. People want to live with their families. Hence, the idea of being isolated brings uncertainty and fear. For example, in Italy, the ‘social contact matrix’ has been considered one reason for the spread of the virus, especially among older people (Pietromarchi 2020). Pietromarchi (2020) also states that there is an intense interaction between the Italian elderly and the younger population compared to other countries. South Africa is, therefore, not unique to this collectivist culture.

Nevertheless, South Africa faces a more significant challenge because it also has a housing crisis, with more than 4 million people living in informal settlements (Royston & Ebrahim 2019; Socio-Economic Rights Institute of South Africa [SERI] 2018). The nature of these informal settlements confronts social distancing and poses barriers to other risk messages and regulations, creating an enormous challenge to mitigating the spread of the virus.

Alcohol consumption was also discouraged through the banning of the sale of alcohol. The South African police minister, Bheki Cele, in a press briefing on 25 March 2020, asked South Africans to stay sober for 21 days (Staff Writer 2020). The government believes that alcohol consumption leads to an increase in trauma patients in hospitals, disrupting the availability of health facilities for coronavirus patients. The consumption of alcohol also reduces people’s adherence to keeping social distance from other people. Therefore, a ban on the sale and distribution of alcohol was effective as of 26 March 2020. The ban was lifted on 01 June 2020 and re-instated on 12 July 2020 (Arthur 2020), effective until 17 August 2020. The next ban, to lessen the cases in trauma units, lasted from 29 December 2020 until 2 February 2021. Based on the WHO’s data collected in 2016, South Africans are the fifth-highest alcohol consumers globally, further classifying South African alcohol consumers as heavy binge drinkers (Staff Writer 2019). However, Wilkinson and Makgato (2016) disputed these conclusions, arguing that there are discrepancies in the WHO’s statistics. Regardless of whether there are discrepancies or not, South Africans alcohol consumption is profuse as was seen from the significant number of panic buyers stocking up on alcohol before and after each ban on alcohol sales (Head 2020a). The establishment of an illegal trade during each ban is also an indication of the drinking habits of South Africans.

The government also placed a ban on public gatherings. Most religious groups viewed this regulation as a provocation. Bishop Bheki Timothy Ngcobo of Nkanyezi Church of Christ defied this call to action and said, ‘This COVID-19 is Satan who is attempting to stop Christians from going to praise God as we wish’ (Hans 2020; Head 2020b; Manyathela 2020). A significant number of pastors shared similar opinions in defiance of this ban. For example, the South African Zionist Church described the ban as Christians’ being oppressed (Hans 2020; Head 2020b; Manyathela 2020). After several consultations between the South African Government and some religious groups and churches, they reached a concession with specific regulations. A maximum of 50 people were allowed at a church service. The need for traditional activities such as initiations also posed as a receptivity barrier to the risk communication process as traditional leaders resisted the ban on initiations and other cultural gatherings.

Globally, COVID-19 regulations brought on political controversies, especially when political leaders made public announcements without scientific backing. For example, Donald Trump and Kevin McCarthy named COVID-19 the ‘Chinese virus’, perpetuating racism (Nichols 2020). In this case, political interference can be perceived as a barrier to risk communication success.

### Combating barriers to receptivity

Disseminating risk messages and imposed regulations were and probably still are the best methods of combating the virus (South African Government News Agency 2020). Empowering the public with information encouraged them to practice necessary precaution measures. However, adherence to the risk messages remains a significant challenge for the South African Government since people continue to get infected with the virus. While the government planned the best ways to communicate the COVID-19 risk information, another task was to encourage the population to change behaviours and to fight the unwillingness to accept change. Simultaneously, the government fought ‘infodemics’, which is defined as an over-abundance of information, some of it accurate and some inaccurate, making it difficult for people to find trustworthy sources and reliable guidance when needed (Zarocostas 2020). Misinformation is the most significant barrier. The government must urge the public only to consume relevant information from credible sources only. In South Africa, only the President, the Minister of Health, and other relevant Ministers were disseminating risk communication messages. These leaders must reach out to other influential leaders on a local and communal level (e.g. church leaders and traditional leaders), to entrust them with further dissemination of information in their communities’ languages. Ultimately, to combat some of the receptivity barriers, the South African Government needs to learn from other countries, for example, Singapore, which imposed its regulations inclusively like imposing the screening of everyone with flu-like symptoms was easy because of the high access to health facilities. Regardless, a combination of risk communication and lawfully enforced regulations prove to be an efficient risk reduction method (South African Government News Agency 2020; WHO 2019).

Important to the study was the need to understand if the government’s and media’s agenda led to behavioural change through the public complying with the risk messages and regulations communicated. Therefore, the ensuing discussion examines a social science theory that relates to the study investigation.

### Agenda-setting Theory: The coronavirus disease 2019 agenda

The media has the power to influence the significance and prevalence of a particular public agenda topic, such as the public awareness of the risks and impact of COVID-19. The AST’ argues that the media shapes how the public should perceive an issue instead of them making their own inferences. This is mainly because of the frequent and prominent coverage of an issue (McCombs & Shaw 1972). As a result, there is a possibility that the public will perceive an aspect essential or not essential based on media coverage or lack thereof. Coronavirus disease 2019 is one such issue that has received wide media coverage and the public has perceived it as a salient issue.

Subsequently, ‘Policy agenda-setting’, ‘Media agenda-setting’, and ‘Public agenda-setting’ are variables that influence the AST (Protess & McCombs 2019). For example, the magnitude and obtrusive nature of COVID-19 influenced the public’s agenda or perception of COVID-19, causing panic and fear. In turn, this led the public to push their own agenda, which in most instances was based on fake news. As a result, the spread of fake news propelled official media channels to sensitise the public to fake news’s harmful impact. Concerning policy agenda-setting, the South African Government imposed a regulation on the prosecution of anyone that creates and spreads fake news (Republic of South Africa 2020d). The agenda of the South African Government was to mitigate the impact and prevent the spread of COVID-19. This agenda influenced the media to frequently report aspects related to the virus, mostly communicating about the risks and impact of COVID-19, the increase in cases, newly imposed regulations, and other issues the government wanted the public to know.

Here we observe a symbiotic relationship amongst these three variables. The media needs official information from the government, and the government requires media coverage on a certain issue, in this case, COVID-19. While the public is the consumer of the information, they are also entitled to know what is happening in their vicinity. Again, the public can also influence what the media will report on. This symbiotic relationship among these variables should be taken note of in risk communication planning.

## Methodology

Respondents in Bloemfontein were selected in a stratified random sampling manner. Stratified random sampling is the division of the entire population into strata groups, and random samples are then selected from each stratum (Hayes & Westfall 2020). With that said, population groups were based on Bloemfontein residential areas and a random sample from each residential area was taken. Bloemfontein’s total population is 556 000 (Statistics South Africa 2020). The study used an application created by Creative Research Systems (2020), and Raosoft (2020), to calculate the sample size. A confidence level of 95% and a margin of error of 5% were used to calculate the sample size out of the population. The anticipated sample size was, therefore 384. However, the study only reached 107 respondents. Most people approached were not interested in the study and it was assumed that people are experiencing COVID-19 information overload. Data saturation was also experienced.

To assess the mitigatory and prevention role of COVID-19 risk communication, a questionnaire was used to collect data. The questionnaire included both open and closed-ended questions. It entailed three sections, the first section covered demographic data with questions on sex, age, education, and residential area. The second section included questions on knowledge and perceptions of COVID-19, for example, a question on the channels the respondents access for COVID-19 information was asked, inter alia. The last section was on attitudes, beliefs, and behaviours including amongst many other questions, a question on if the respondent practices the regulations communicated. A questionnaire was administered through WhatsApp, telephone, and emails to adhere to the COVID-19 regulations of conforming to social distancing. Some respondents could fill in the questionnaire personally, and some were assisted.

### Ethical considerations

Ethical clearance was obtained from the Ethics Committee of the Faculty of Natural and Agricultural Sciences, the University of the Free State with reference number: UFS-HSD2020/1303/1609.

## Results and discussions

**FIGURE 1:**
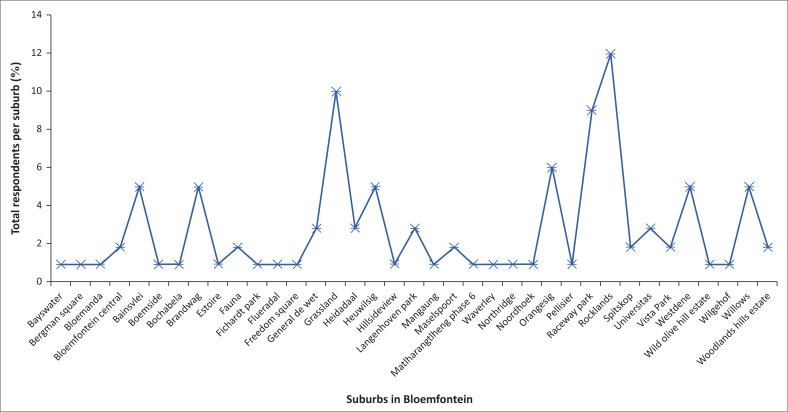
Respondents per suburb in Bloemfontein.

Of the 107 respondents that participated in the study, the majority (63%) were females, and the rest (37%), males. Their age groups ranged from 19–29 (29%), 30–41 (50%), 42–53 (14%), and over 54 (7%). Most of the participants (83%) had tertiary education, 10% had vocational training, and the rest (7%) had high school education. In January 2021, Mangaung Metropolitan Municipality (MMM) had 29 069 COVID-19 cases, the highest in the Free State Province. Out of the eight towns in MMM, Bloemfontein had the highest number (22 672) number of reported COVID-19 cases (Department of Health, Free State Province 2021). Bloemfontein’s COVID-19 status also made it the ideal study area to investigate the study objectives. From the respondents, a small number (7%) of the participants indicated that they had tested positive for COVID-19. Figure 1 illustrates the suburbs in which the respondents reside and the respondents’ total number per suburb.

The study obtained respondents from 25 residential areas out of an estimated 92 suburbs (Property24 2020). Figure 1 further shows a reasonable number of respondents from each of the 25 residential areas. Therefore, the researchers believe that they could obtain a fair perception of what was happening in Bloemfontein regarding COVID-19.

**FIGURE 2:**
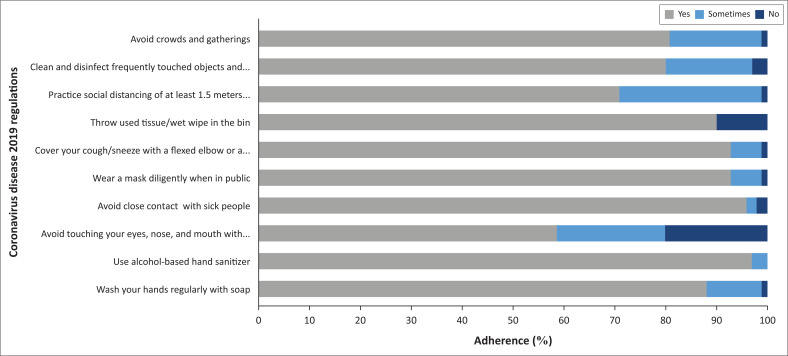
Coronavirus disease 2019 regulations adherence.

Figure 2 shows the level of adherence to the regulations communicated to prevent and mitigate the spread of COVID-19. Adherence to the regulations communicated by the government is significant, with an average of 85%. This is an indication that the government’s ‘Policy agenda’ was successful and through the ‘Media agenda’, the government was able to influence the respondents’ risk behaviour. An average of 12% of the respondents indicated that they sometimes adhere to the regulations and a low 3% stated that they do not practice the regulations. To support the 12% and 3% adherence numbers, the respondents argued that they are forgetful, especially around family and close friends, struggle to break old habits like touching one’s face, and certain circumstances do not allow for regulation adherence (like keeping the 1.5-m distance from others in a shopping area). During risk communication, some people do not have the ability and are unwilling to receive the information communicated, and therefore, not able to adapt to the new ideas communicated. Also, if the public’s agenda is negative, then the receptivity of information communicated might be affected.

An inference made from Figure 2 is that not touching one’s face is problematic for most people. People also struggle to maintain the social distance of 1.5 m. Nearly 98% of the respondents use sanitiser and avoid contact with sick people. Most respondents also indicated that they wear masks in public, and most of them cover their mouth and nose with a flexed elbow when coughing. Problematic is that 12% of the respondents do not, or only sometimes, wash their hands with soap and water. Klöckner and Prugsamatz (2012) argue that old habits stand in the way of changing behaviour. This in turn affects the risk communication goals.

**FIGURE 3:**
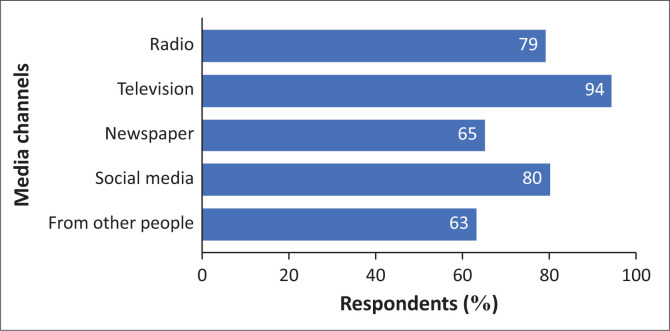
Media channels where respondents obtain coronavirus disease 2019 information.

In risk communication, the media channels used to distribute risk messages are important (Reynolds & Seeger 2005). Also, media is a very important stakeholder in risk communication and disaster risk reduction (UNISDR 2015). Therefore, the study investigated the media channels from which respondents obtain COVID-19 information (See Figure 3). A significant number of the respondents (94%) obtain their information from television. The reason for this high reliance on television is because it is a trusted source of information and because the President of South Africa, as a credible public figure, appeared regularly on television to make live announcements about COVID-19. Accordingly, Mkhondo (2020) identified President Ramaphosa as a bold, assertive, courageous, and decisive leader during the pandemic compared to other world leaders. This shows that the governments ‘Policy agenda’ was working.

**FIGURE 4:**
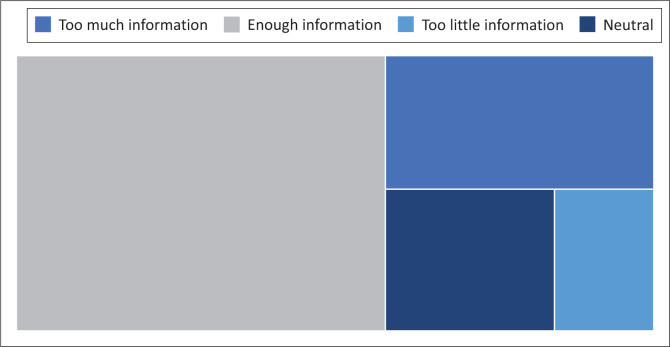
Perceptions regarding the amount of information about coronavirus disease 2019 shared in the media.

Ever since the COVID-19 pandemic, various media channels have frequently aired information about the virus. As shown in Figure 4, at least 15% of the respondents thought they received too much information, although the majority (69%) perceived the information to be enough. A small number (6%) believed the information was too little. About 10% of the respondents did not answer this question. The small percentage of the respondents who did not answer could mean that they probably do not follow the news on COVID-19 or that they were experiencing news fatigue. News fatigue is when one becomes tired of the constant negativity or airing of a certain issue (Urban Dictionary 2020). Since most of the respondents perceived the information received as enough, the issue of overwhelming information is possibly minimal and not perceived as problematic by the Bloemfontein public.

Each community has influential people or organisations. If these influential people are credible and trustworthy, they can influence behaviours that could save lives (Reynolds 2005). An influential person is someone whose actions and opinions strongly influence the course of events. Therefore, the researchers asked the respondents to share their perceptions of influential people and COVID-19. It seemed as if influential people do not impact swaying behaviours regarding the spread of the virus, with 36% of the respondents answering ‘yes’, they will take extra care of themselves if an influential person speaks about COVID-19 precautions. Only 28% of the respondents said they might be influenced, while 36%, said they would not be influenced. In South Africa, celebrities with a huge following and with an ability to influence their followers, took to social media to help curb the spread of the virus (Rakhetsi 2021; Schimke 2020). From the ‘media agenda setting’ perspective, the media does explore influential people to support their agenda on a particular issue. Social media (like Twitter and Facebook) also has a significant number of influential people who explore social media platforms to push a certain agenda.

Since the President of South Africa and his Ministers were the primary official COVID-19 spokespersons, the study investigated how the respondents perceived them and the messages they disseminated. As stated earlier, in risk communication, the messages and strategies communicated must be trustworthy, acceptable, feasible, and useful. The question, ‘Do you trust the following government official/s when he/she is communicating about COVID-19?’, was asked and the options, ‘yes’, ‘maybe’, ‘no’ were provided. Among the Ministers, the most trusted person was the Health Minister (79%). The respondents who indicated that they do not trust the Ministers believed that their messages were pessimistic instead of encouraging. The respondents were also of the opinion that the untrusted Ministers are political with hidden interests, contradict each other, and are corrupt. Therefore, the respondents view the officials as untrustworthy and uninformed. And that they were not advised by experts, therefore, unknowledgeable. They also miscommunicated and were driven by fear and not facts, and that they lie and lack concern for the country’s citizens. ‘Dr Zweli Mkhize, the Health Minister, tested positive, so that says a lot’, said one respondent. In this case, the respondent is probably doubting what the Minister of Health communicates since he contracted the virus.

**FIGURE 5:**
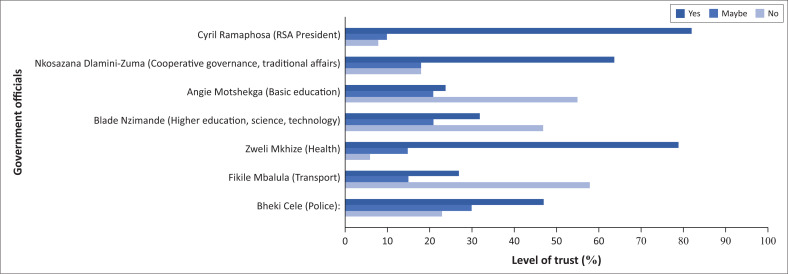
Trust in the government officials when communicating about coronavirus disease 2019.

As shown in Figure 5, the most trusted person (82%), is the President. This corroborates with Mkhondo’s (2020) thinking that the President is assertive in his communication, making him a credible and trustworthy COVID-19 spokesperson.

The disaster management centre under the Ministry of CoGTA is leading the response and management of COVID-19. Hence, this study investigated the respondents’ knowledge of the Mangaung Metropolitan Disaster Management Centre. It also investigated if the respondents received information about COVID-19 from the local disaster management centre. At the local level, the disaster management centre is supposed to monitor the implementation of regulations and ensure compliance like the number of people allowed at funerals. They are also supposed to make available resources to help fight the virus and many other activities. Just more than a third of the respondents (37%) said they knew that there is a local disaster management centre, while more than half (63%) are unaware of the institution. Less than 8% of the respondents indicated that they received information about COVID-19 from the local disaster management centre. It seems that the disaster management centre is silent and inactive regarding COVID-19 risk communication at the local level. As noted in the AST, policy agenda-setting is when the government takes action in response to a certain matter. However in this case, it seems the Mangaung Metropolitan Disaster Management Centre was not visible in its response to COVID-19 at a local level. Perhaps the public and the media did not place much influence on the disaster management centre for it and its work to be noticeable.

The final question was whether the respondents perceive the Bloemfontein population as compliant with the COVID-19 regulations.

**FIGURE 6:**
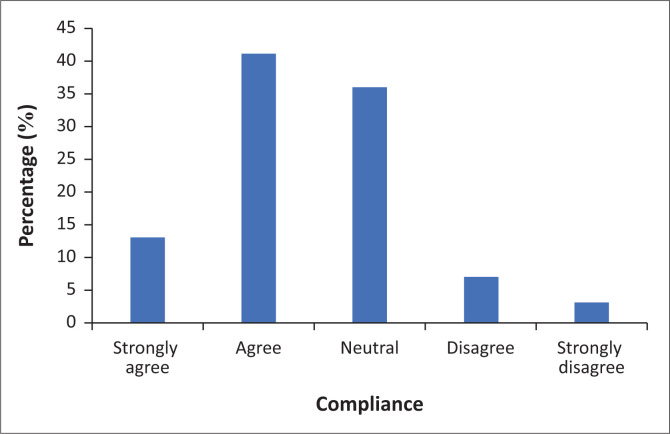
Bloemfontein population compliancy to coronavirus disease 2019 regulations.

Figure 6 illustrates the respondents’ perceptions regarding the Bloemfontein population’s compliance with COVID-19 regulations. At least 13% of the respondents ‘strongly agreed’ and 41% ‘agreed’ that the Bloemfontein population was compliant. This means that as observed by the respondents, the Bloemfontein population is listening and adhering to the risk communication messages and regulations indicating success in the policies and media’s agenda. While 36% remained neutral in their response, 7% disagreed, and 3% strongly disagreed that the Bloemfontein population was compliant. The 36% response in the respondents indicating neutrality could mean that as they have observed the Bloemfontein population, sometimes the people abide and sometimes they do not. Perhaps this was confusing for the respondents and they were unable to conclude if the Bloemfontein population was compliant or not. The study, therefore, concluded that the respondents perceive the Bloemfontein population as a compliant population. The questionnaire probed the respondents’ general perceptions concerning COVID-19 communication, which are discussed in the ensuing section.

### Respondents general perceptions

The respondents indicated that there is poor communication between the Mangaung Metropolitan Disaster Management Centre and the Bloemfontein community at the local level. These respondents would expect the local disaster management centre to be visible because the *Disaster Management Act*, 57 of 2002 was used to declare the virus a disaster and the fact that disaster management is the coordinating function. Research by Wiggill (2013) revealed that local-level disaster risk communication mechanisms are non-existent in South Africa (Van Niekerk 2014). Therefore, at the local level, the media and the government must continue driving the COVID-19 risk communication agenda through community radios, local newspapers and through traditional methods. An increase in awareness campaigns in Bloemfontein, especially in local languages and use of traditional communication methods are needed, stated the respondents. The use of a local language makes the risk message easy to understand and also the population feels that the risk communicator is solicitous. Traditional methods of communication like regular loud-hailers or billboards in the residential areas might help with full access to the risk messages.

Also, the visibility of the disaster management centre, leading the dissemination of information at the local level as a recognised COVID-19 response organisation might reduce some receptivity barriers. The respondents argued that the government should inform the communities about the institutions that play a significant role in their lives, with specific reference to the disaster management centre. The reason for this request could be because they will be able to report issues of non-compliance or requests for resources. Some of the responsibilities of the disaster management centre are to obtain resources during a disaster and monitor compliance to regulations imposed (Section 27(2)(a) of the *Disaster Management Act*).

The respondents feel that although people might adhere to the regulations, they will still be infected. The respondents feel like this because adherence to the regulations is not possible 100%. People tend to relax when they are with people they trust (family, friends, and colleagues), thereby disregarding social distancing, or perhaps certain situations does not allow adherence to the regulations, like when grocery shopping, inter alia.

Furthermore, the respondents feel that the government should enforce more stringent laws on crowd gathering, that the wearing of a face shield alone without a mask should be prohibited. Medical experts believe that a plastic face shield is inadequate for protecting oneself from the virus (Cesak 2020). The respondents believe that the government should show the public the effects of COVID-19, just like they did with HIV and/or AIDS. Unfortunately, for this respondent scare tactics have been proven to not work anymore (Columbia University’s Mailman School of Public Health 2015). A more non-sensitive and scientific approach might be the best approach in risk communication.

## Conclusion

Coronavirus disease 2019 risk communication included the dissemination of risk reduction messages and risk reduction regulations. To investigate the mitigatory and preventative role COVID-19 risk communication played, the study assessed, inter alia, the Bloemfonteins’ population attitude, beliefs and behaviours towards the risk messages and regulations. The study found that although most participants indicated that they use sanitiser and stay clear of sick people, problematic aspects are that they struggle to keep the social distance of 1.5 m and do not wash their hands regularly with soap and water. Besides some other barriers to receptivity, most respondents’ social-economic status can be an indication of why they struggle with these aspects. This is especially in densely populated informal settlements and high-density residential areas like Bergman Square, Freedom Square, Phase 6, Mangaung, etc. (see Figure 1), where they have limited access to water and proper sanitation facilities.

From this study, it can be deduced that receptivity barriers remain a challenge wherever there is risk communication on or about COVID-19. However, imposing regulations and access to resources that accompany the dissemination of the risk messages might be the best approach. At least based on the significant number of the respondents who adhere to the regulations communicated, the study deduces that COVID-19 risk communication is successful. The media agenda and policy agenda are playing a positive role in influencing behaviour change. However, the public agenda especially the spread of fake news seems to affect the government’s efforts in mitigating and preventing the spread of the virus.

The South African Government through the influence of the media should also encourage people to start adapting to the COVID-19 risk communication messages. And to take them as a new way of life. Through risk communication, the media must preach the theme ‘living with COVID-19’. This theme comes with the permanent enforcement of the currently communicated COVID-19 protection measures (vaccinating, wearing masks, social distancing, sanitising, and handwashing), which can become the new norm and global culture. This can be until a certain time that the virus has ended.

Regardless of the role that the disaster management centres are supposed to play in responding to COVID-19, the study found that the local Mangaung Metropolitan Disaster Management Centre was not known by the respondents. It is therefore imperative for the disaster management centre to be more proactive, visible and assertive in their risk reduction activities and communication with the public. Knowledge of the local disaster management centre will assist people to know where to report non-compliance, and request for resources required to mitigate the spread of the virus. At the local level, the centre can further advocate through influential stakeholders the adherence to risk communication strategies imposed by the government. Risk communication should be pivotal in all disaster management operations and deserves the bigger part of the disaster management operations’ financial investment.

## Footnotes

1 Designated under section 3, 27(1)(2) of the *Disaster Management Act*, which authorises a Minister as a Cabinet member designated by the President to declare a national disaster.

2 It is important to note that the comparison made of these countries’ strategies is limited to April and March 2020, when COVID-19 started showing its vicious potential of spreading like wildfire.
